# Complete chloroplast genome sequence data of *Camellia handelii* Sealy (Theaceae)

**DOI:** 10.1016/j.dib.2026.113144

**Published:** 2026-08-06

**Authors:** Zhun Xiao, Jinrui Yan

**Affiliations:** a Changsha Social Work College, Changsha, Hunan, 410004, China; b Central South University of Forestry and Technology, Changsha, Hunan, 410004, China

**Keywords:** *Camellia handelii*, Chloroplast genome, Phylogenetic analysis

## Abstract

This data article describes the complete chloroplast genome sequence data of *Camellia handelii* Sealy, a fragrant-flowered species of *Camellia* sect. *Theopsis*. Fresh young leaves were collected from a cultivated individual in Changsha, China. Genomic DNA was extracted and sequenced on the Illumina NovaSeq platform to generate 100 bp paired-end reads. Raw reads were processed using fastp, and chloroplast-derived reads were enriched by mapping to the reference chloroplast genome of *Camellia sinensis*. The chloroplast genome was assembled using GetOrganelle and annotated using GeSeq.

The complete chloroplast genome of *C. handelii* is 156,901 bp in length and has a typical quadripartite structure, including an LSC region of 86,505 bp, an SSC region of 18,268 bp, and two IR regions of 26,064 bp each. The genome has an overall GC content of 37.33% and contains 132 annotated genes. A phylogenetic dataset based on 72 concatenated orthologous single-copy chloroplast genes from 44 taxa was also generated. The genome sequence, raw sequencing reads, and phylogenetic dataset are available under GenBank accession PZ504125, BioProject PRJNA1476376, BioSample SAMN60712287, SRA accession SRR39069759, and Figshare DOI https://doi.org/10.6084/m9.figshare.32625783.

Specifications Table

**Table:** 

Subject	Biology
Specific subject area	Omics: Chloroplast genomics
Type of data	Table, Figure, Phylogenetic tree Raw, Filtered, Analyzed, Processed
Data collection	Fresh young leaves of *Camellia handelii* were collected from a cultivated individual and used for total genomic DNA extraction. A short-read library was constructed and sequenced on the Illumina NovaSeq platform to generate 100-bp paired-end reads. Raw reads were processed using fastp. Chloroplast-derived reads were enriched by mapping to the *Camellia sinensis* chloroplast genome, assembled using GetOrganelle, annotated using GeSeq, and visualized using CPStools. Phylogenetic analysis was performed using PhyloSuite, MAFFT, IQ-TREE and iTOL.
Data source location	Institution: Camellia Garden of Central South University of Forestry and Technology City/Town/Region: Changsha, Hunan Province Country: China Latitude and longitude: 28°08′14″N, 112°59′27″E Voucher information: The voucher specimen of *Camellia handelii* was deposited at the State Key Laboratory of Woody Oil Resource Utilization, Central South University of Forestry and Technology, under voucher number 20250418CS-1. Contact person: Zhun Xiao; email: xz1271088@163.com.
Data accessibility	Repository name: NCBI Direct URL to data of complete chloroplast genome and raw data. 1) https://www.ncbi.nlm.nih.gov/nuccore/PZ504125 2) https://www.ncbi.nlm.nih.gov/bioproject/PRJNA1476376 3) https://www.ncbi.nlm.nih.gov/biosample/SAMN60712287 4) https://www.ncbi.nlm.nih.gov/sra/SRR39069759 Repository name: Figshare Direct URL to the associated phylogenetic and assembly-validation data https://doi.org/10.6084/m9.figshare.32625783
Related research article	None.

## 1 Value of the Data

• The complete chloroplast genome sequence of *Camellia handelii* provides a new chloroplast genome resource for *Camellia* sect. *Theopsis* and the family Theaceae. These data can be used as a reference for chloroplast genome comparison, genome structure analysis, and organelle genomic studies of related *Camellia* species.
• The dataset includes the assembled chloroplast genome sequence, genome annotation files, and related sequence information. These data can be reused for gene annotation comparison, identification of chloroplast coding regions, and comparative analyses of genome structure and gene content among *Camellia* taxa.
• These data may serve as a genomic resource for future molecular identification and taxonomic studies of *C. handelii* and related *Camellia* species, including wild, ornamental and fragrant-flowered germplasm.
• The phylogenetic dataset generated from 72 concatenated orthologous single-copy chloroplast genes can be integrated with published chloroplast genome datasets for broader phylogenomic analyses of *Camellia* and related Theaceae taxa.
• The raw Illumina sequencing reads can be reused for chloroplast genome reassembly, assembly validation, and methodological comparison of chloroplast genome assembly and annotation workflows.

## 2 Background

The genus *Camellia* L. belongs to the family Theaceae and includes many species with important economic, ornamental, and ecological value [1]. Several *Camellia* species are widely used as tea plants, oil-tea crops, ornamental shrubs, and garden plants. Complete chloroplast genomes are useful genomic resources for plant molecular identification, chloroplast genome comparison, and phylogenetic analyses because they provide information on genome structure, gene content, and sequence variation among related taxa [2].

**Fig. 1:**
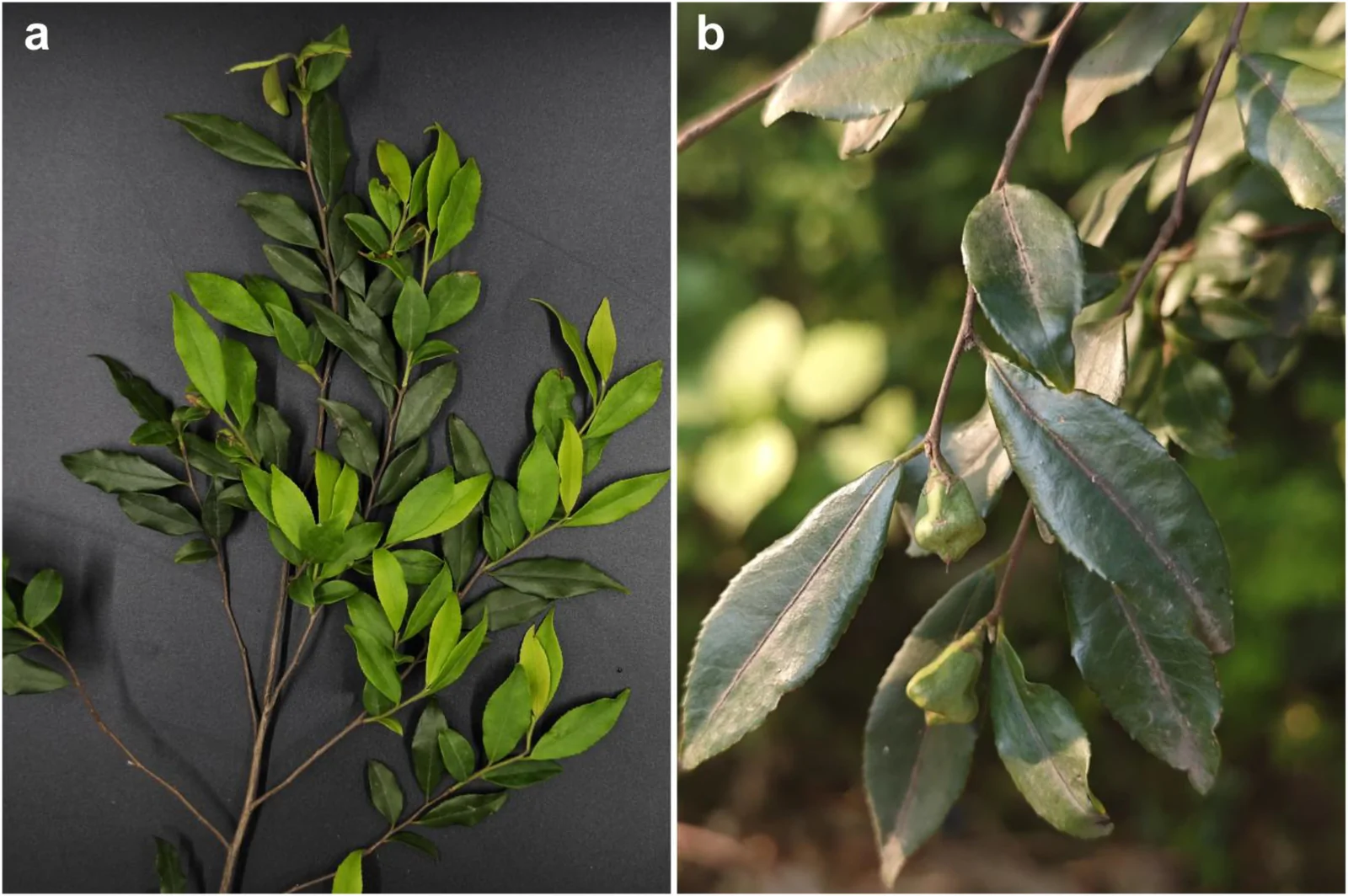
Morphological features of *C. handelii*. (a) Leafy branch. (b) Fruit-bearing branch.

*Camellia handelii* Sealy is an evergreen shrub of *Camellia* sect. *Theopsis (*Fig. 1). It is native to China and is distributed in Guangxi, Hunan, Hubei, and Sichuan [3]. This species has abundant and fragrant flowers, strong adaptability, compact plant architecture, and attractive foliage, making it a potentially valuable ornamental resource and one of the rare fragrant-flowered species in *Camellia* [3,4]. *C. handelii* is also considered a potential bioenergy tree resource; its seed oil can be used for energy production and in industrial, medical, and cosmetic applications [5]. Sect. *Theopsis* comprises 49 species [1]. However, at the time of manuscript preparation, complete chloroplast genome records in the NCBI database were available for only a few species assigned to this section, including *C. costei, C. cuspidata, C. elongata*, and *C. fraterna*. Therefore, the chloroplast genome dataset presented here was generated to provide reusable genomic information for chloroplast genome comparison, species identification, molecular marker development, and phylogenomic studies of *C. handelii* and related *Camellia* species.

## 3 Data Description

**Fig. 2:**
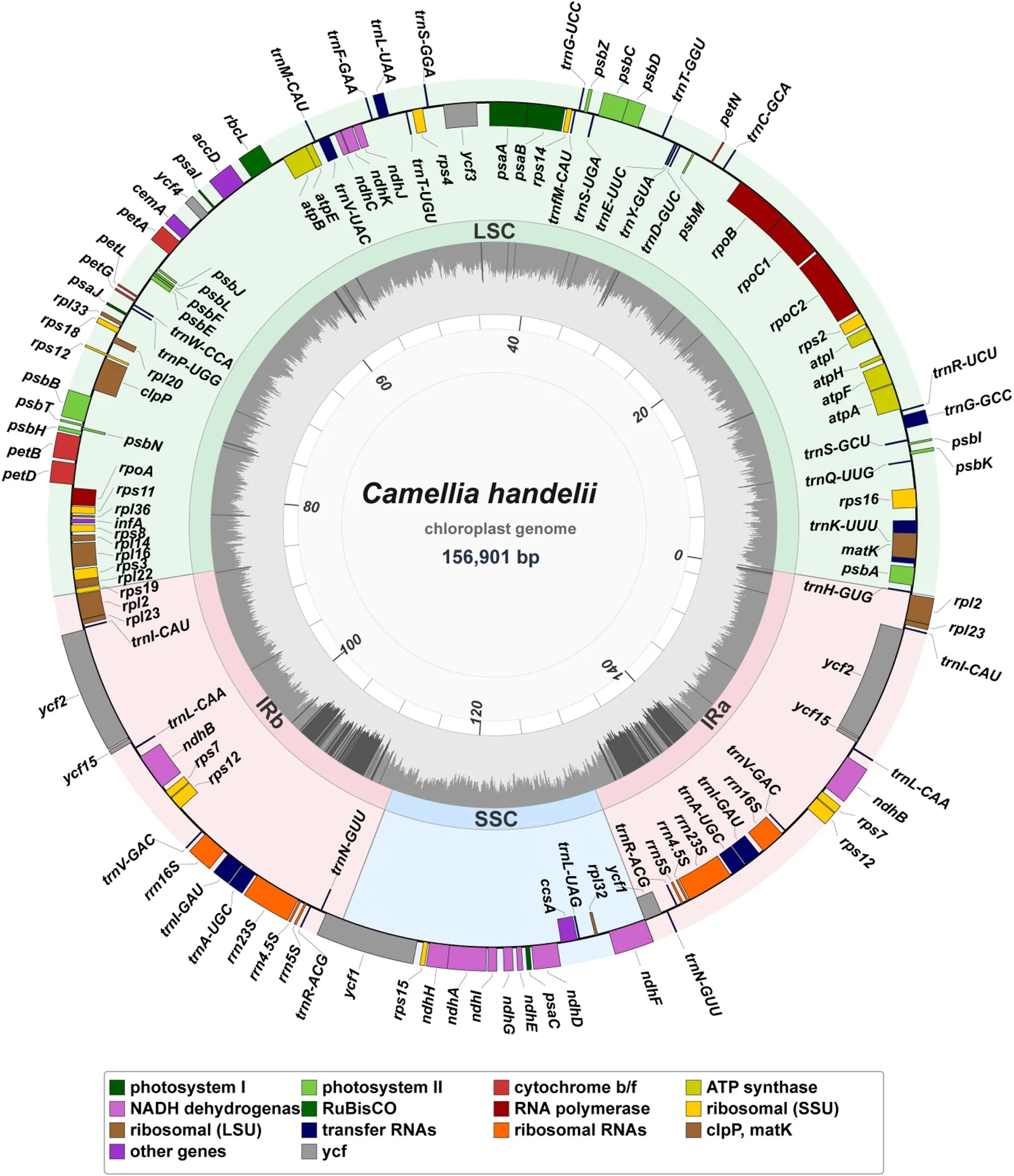
Circular map of the complete chloroplast genome of *C. handelii*. Genes shown outside and inside the outer circle are transcribed clockwise and counterclockwise, respectively. Genes are color-coded according to their functional categories. The inner gray histogram represents GC content across the chloroplast genome. LSC, large single-copy region; SSC, small single-copy region; IRa and IRb, inverted repeat regions.

The complete chloroplast genome of *C. handelii* is a circular DNA molecule of 156,901 bp in length. It shows the typical quadripartite structure of angiosperm chloroplast genomes, consisting of a large single-copy region (LSC) of 86,505 bp, a small single-copy region (SSC) of 18,268 bp, and a pair of inverted repeat regions (IRa and IRb) of 26,064 bp each (Fig. 2). The overall GC content of the chloroplast genome is 37.33%. The GC contents of the LSC, SSC, IRa, and IRb regions are 35.35%, 30.58%, 42.98%, and 42.98%, respectively.

Of the clean paired-end reads, 1170,646 reads mapped to the final chloroplast genome, corresponding to a mapping rate of 1.17%. Among the primary mapped reads, 1152,526 reads (98.45%) were properly paired. All 156,901 bp of the assembled chloroplast genome were covered by sequencing reads, resulting in 100% genome coverage and a mean sequencing depth of 740.47×. The detailed per-base depth and read-mapping statistics are available in the associated Figshare dataset.

A total of 132 genes were annotated in the *C. handelii* chloroplast genome. These annotated genes were assigned to three functional categories: photosynthesis-related genes, self-replication-related genes, and other genes (Table 1). The photosynthesis-related genes include genes encoding subunits of photosystem I, photosystem II, NADH dehydrogenase, the cytochrome b/f complex, ATP synthase, and the large subunit of Rubisco. The self-replication-related genes include genes encoding large and small ribosomal subunit proteins, RNA polymerase subunits, ribosomal RNAs, and transfer RNAs. Other annotated genes include *matK, clp*P, *cem*A, *acc*D, *ccs*A, *inf*A, and conserved open reading frame genes.

**Table 1:** Gene composition and functional classification of the *C. handelii* chloroplast genome.

Category	Gene Group	Gene Name	Number
Photosynthesis	Subunits of photosystem I	*psa*B, *psa*A, *psa*I, *psa*J, *psa*C	5
	Subunits of photosystem II	*psb*A, *psb*K, *psb*I, *psb*M, *psb*D, *psb*C, *psb*Z, *psb*J, *psb*L, *psb*F, *psb*E, *psb*B, *psb*T, *psb*N, *psb*H	15
	Subunits of NADH dehydrogenase	*ndh*J, *ndh*K, *ndh*C, *ndh*B(2)#, *ndh*F, *ndh*D, *ndh*E, *ndh*G, *ndh*I, *ndh*A#, *ndh*H	12
	Subunits of cytochrome b/f complex	*pet*N, *pet*A, *pet*L, *pet*G, *pet*B#, *pet*D#	6
	Large subunit of rubisco	*rbc*L	1
	Subunits of ATP synthase	*atp*A, *atp*F#, *atp*H, *atp*I, *atp*E, *atp*B	6
Self-replication	Proteins of large ribosomal subunit	*rpl*33, *rpl*20, *rpl*36, *rpl*14, *rpl*16#, *rpl*22, *rpl*2(2)#, *rpl*23(2), *rpl*32	11
	Proteins of small ribosomal subunit	*rps*12(2)##, *rps*16#, *rps*2, *rps*14, *rps*4, *rps*18, *rps*11, *rps*8, *rps*3, *rps*19, *rps*7(2), *rps*15	14
	Subunits of RNA polymerase	*rpo*C2, *rpo*C1#, *rpo*B, *rpo*A	4
	Ribosomal RNAs	*rrn*16S(2), *rrn*23S(2), *rrn*4.5S(2), *rrn*5S(2)	8
	Transfer RNAs	*trn*H-GUG, *trn*K-UUU#, *trn*Q-UUG, *trn*S-GCU, *trn*G-GCC#, *trn*R-UCU, *trn*C-GCA, *trn*D-GUC, *trn*Y-GUA, *trn*E-UUC, *trn*T-GGU, *trn*S-UGA, *trn*G-UCC, *trn*fM-CAU, *trn*S-GGA, *trn*T-UGU, *trn*L-UAA#, *trn*F-GAA, *trn*V-UAC#, *trn*M-CAU, *trn*W-CCA, *trn*P-UGG, *trn*I-CAU(2), *trn*L-CAA(2), *trn*V-GAC(2), *trn*I-GAU(2)#, *trn*A-UGC(2)#, *trn*R-ACG(2), *trn*N-GUU(2), *trn*L-UAG	37
Other genes	Maturase	*mat*K	1
	Protease	*clp*P##	1
	Envelope membrane protein	*cem*A	1
	Acetyl-CoA carboxylase	*acc*D	1
	c-type cytochrome synthesis gene	*ccs*A	1
	Translation initiation factor	*inf*A	1
	Conserved open reading frames	*ycf*3##, *ycf*4, *ycf*2(2), *ycf*15(2), *ycf*1	7
Total			132

Note: “#” and “##” indicate genes containing one and two introns, respectively; “(n)” indicates the number of gene copies.

Several genes were present in two copies, mainly because of their location in the inverted repeat regions. These duplicated genes include *ndh*B, *rpl*2, *rpl*23, *rps*12, *rps*7, *trn*I-CAU, *trnL*-CAA, *trn*V-GAC, *trn*I-GAU, *trn*A-UGC, *trn*R-ACG, *trn*N-GUU, *rrn*16S, *rrn*23S, *rrn*4.5S, *rrn*5S, *ycf*2, and *ycf*15. Intron-containing genes were also identified in the chloroplast genome. Genes containing one intron include *ndh*B, *ndh*A, *pet*B, *pet*D, *atp*F, *rpl*16, *rpl*2, *rps*16, *rpo*C1, *trn*K-UUU, *trn*G-GCC, *trn*L-UAA, *trn*V-UAC, *trn*I-GAU, and *trn*A-UGC. Genes containing two introns include *rps*12, *clp*P, and *ycf*3.

**Fig. 3:**
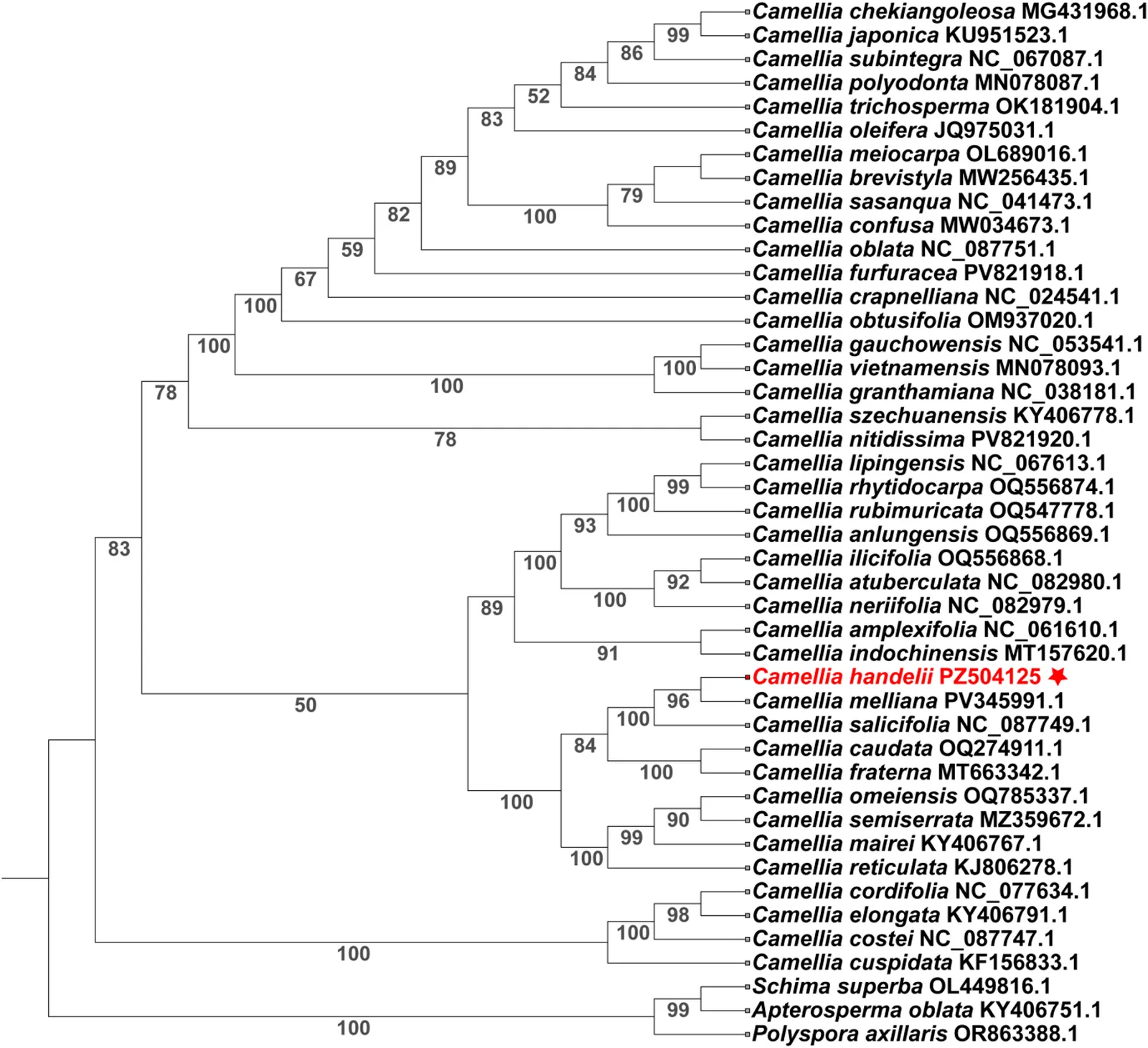
Maximum-likelihood phylogenetic tree of *C. handelii* and related Theaceae species inferred from 72 concatenated orthologous single-copy chloroplast genes. *C. handelii* is highlighted, and numbers on branches indicate node support values.

The phylogenetic dataset was constructed from 72 concatenated orthologous single-copy chloroplast genes from 44 taxa. The resulting maximum-likelihood tree placed *C. handelii* within the *Camellia* clade, where it formed a sister relationship with *C. melliana* with high node support. The *C. handelii*–*C. melliana* clade was further grouped with *C. salicifolia, C. caudata*, and *C. fraterna (*Fig. 3).

## 4 Experimental Design, Materials and Methods

### 4.1 Plant material

Samples were collected from the Camellia Garden of Central South University of Forestry and Technology, Changsha, China (28°08′14″N, 112°59′27″E). Fresh young leaves were collected from a healthy and vigorously growing individual of *C. handelii* and used for genomic DNA extraction. The voucher specimen of *C. handelii* was deposited at the State Key Laboratory of Woody Oil Resource Utilization, Central South University of Forestry and Technology (contact person: Zhun Xiao; email: xz1271088@163.com) under the voucher number 20250418CS-1. The chloroplast genome dataset reported in this article was generated from a single cultivated individual of *C. handelii*.

### 4.2 DNA extraction and sequencing

Total genomic DNA was extracted from fresh young leaves of *C. handelii* using the DNeasy Plant Mini Kit (QIAGEN, Hilden, Germany) following the manufacturer’s protocol. The quality of the extracted DNA was assessed before library construction. DNA degradation and potential contamination were examined by 1% agarose gel electrophoresis, DNA purity was evaluated using a NanoPhotometer spectrophotometer, and DNA concentration was quantified using a Qubit fluorometer.

Qualified genomic DNA was used for short-read library construction. Briefly, genomic DNA was fragmented into suitable sizes, followed by end repair, 3′ A-tailing, adapter ligation, fragment size selection, purification, and PCR enrichment. After library construction, the library concentration was preliminarily quantified using a Qubit fluorometer, and the insert size distribution was examined using an Agilent 2100 Bioanalyzer. The effective library concentration was further quantified by quantitative PCR to ensure that the library met the requirements for sequencing. The qualified library was then sequenced on the Illumina NovaSeq platform to generate 100-bp paired-end raw reads.

Raw sequencing reads were processed using fastp [6]. Adapter-contaminated reads, reads containing a high proportion of ambiguous bases, and low-quality reads were removed during read preprocessing. The resulting clean reads were used for chloroplast read enrichment, genome assembly, and subsequent analyses.

### 4.3 Assembly and annotation of the chloroplast genome

Approximately 10 Gb of paired-end clean reads were first mapped to the reference chloroplast genome of *Camellia sinensis* (NC_061691.1) using BWA-MEM [7] to enrich chloroplast-derived reads. The enriched chloroplast reads were then assembled into a circular chloroplast genome using GetOrganelle v1.7.7.1 [8] with the following parameters: “-R 15 -k 21,35,45,55,65,75,85,95 -F embplant_pt -t 5″.

To evaluate assembly quality, the paired-end clean reads were mapped back to the final chloroplast genome using BWA-MEM [7]. Read-mapping statistics, genome coverage and sequencing depth were calculated using SAMtools v1.21 [9]. The read alignments were examined to confirm continuous coverage across the assembled genome.

Chloroplast genome annotation was performed using the online GeSeq platform [10]. The junctions between the LSC, SSC, IRa and IRb regions were manually inspected. Annotation quality was further verified by manually checking gene boundaries, start and stop codons, exon–intron junctions, duplicated genes located in the inverted repeat regions and the trans-spliced gene *rps*12. Standardized annotation files in GenBank and GFF3 formats were then generated. The circular chloroplast genome map was drawn using CPStools [11]. The complete chloroplast genome of *C. handelii* has been deposited in GenBank (https://www.ncbi.nlm.nih.gov/genbank/) under the accession number PZ504125. The associated BioProject, SRA, and BioSample numbers are PRJNA1476376, SRR39069759, and SAMN60712287, respectively.

### 4.4 Phylogenetic analysis

To determine the phylogenetic position of *Camellia handelii* based on chloroplast genome data, 43 complete chloroplast genomes were downloaded from the GenBank database, including 40 *Camellia* species and three outgroups: *Schima superba* (OL449816.1), *Apterosperma oblata* (KY406751.1), and *Polyspora axillaris* (OR863388.1). Together with the newly assembled chloroplast genome of *C. handelii*, a total of 44 chloroplast genomes were used for phylogenetic analysis. PhyloSuite was used to extract 72 orthologous single-copy genes from these chloroplast genomes [12]. The extracted genes were concatenated in the same gene order across all samples to generate the final phylogenetic dataset.

Multiple sequence alignment was performed using MAFFT [13]. The maximum-likelihood (ML) phylogenetic tree was constructed using IQ-TREE [14]. The best-fit nucleotide substitution model was automatically selected by ModelFinder, and TVM+F+I was identified as the optimal model. Node support was assessed using 1000 ultrafast bootstrap replicates and the SH-like approximate likelihood-ratio test (SH-aLRT). The resulting ML tree was exported in Newick format and visualized and edited using the online iTOL platform [15].

## Limitations

This dataset was generated from a single cultivated individual of *C. handelii* and therefore represents only the chloroplast genome of that individual. It does not capture the full range of intraspecific chloroplast genome variation among different individuals or populations. In addition, the dataset is limited to chloroplast genomic information and does not include nuclear or mitochondrial genome data. Additional chloroplast genomes from multiple individuals and populations, together with nuclear and mitochondrial genomic datasets, would be required for population-level analyses and for investigating complex evolutionary processes such as hybridization and introgression.

## Ethics Statement

All authors have read and followed the ethical requirements for publication in *Data in Brief*. This work does not involve human subjects, animal experiments, or any data collected from social media platforms. The plant material of *Camellia handelii* used in this data article was collected from cultivated plants and did not involve endangered or protected wild plant sampling.

## CRediT Author Statement

**Jinrui Yan:** Conceptualization, Methodology, Investigation, Resources, Writing – review & editing. **Zhun Xiao:** Software, Formal analysis, Data curation, Visualization, Writing – original draft, Writing – review & editing.

## Data Availability

Chloroplast genome assembly validation and phylogenetic data for Camellia handelii and related Theaceae species (Original data)
NCBI Sequence Read ArchiveRaw Illumina sequencing reads of Camellia handelii (Original data)
NCBI GenBankComplete chloroplast genome sequence data of Camellia handelii Sealy (Original data)
